# Analyzing Key Factors on Training Days within a Standard Microcycle for Young Sub-Elite Football Players: A Principal Component Approach

**DOI:** 10.3390/sports12070194

**Published:** 2024-07-16

**Authors:** José Eduardo Teixeira, Luís Branquinho, Ricardo Ferraz, Ryland Morgans, Samuel Encarnação, Joana Ribeiro, Pedro Afonso, Nemat Ruzmetov, Tiago M. Barbosa, António M. Monteiro, Pedro Forte

**Affiliations:** 1 Department of Sports Sciences, Polytechnic of Guarda, 6300-559 Guarda, Portugal; 2 Department of Sports Sciences, Polytechnic Institute of Bragança, 5300-252 Bragança, Portugal; samuel01.encarnacao@gmail.com (S.E.); barbosa@ipb.pt (T.M.B.); mmonteiro@ipb.pt (A.M.M.); pedromiguel.forte@iscedouro.pt (P.F.); 3 SPRINT—Sport Physical Activity and Health Research & Innovation Center, 6300-559 Guarda, Portugal; 4 Research Center in Sports, Health and Human Development, 6201-001 Covilhã, Portugal; luis.branquinho@iscedouro.pt (L.B.); ricardompferraz@gmail.com (R.F.); pmvafonso@gmail.com (P.A.); 5 LiveWell—Research Centre for Active Living and Wellbeing, Polytechnic Institute of Bragança, 5300-252 Bragança, Portugal; 6 CI-ISCE, ISCE Douro, 4560-547 Penafiel, Portugal; joana.ribeiro@iscedouro.pt; 7 Biosciences Higher School of Elvas, Polytechnic Institute of Portalegre, 7300-110 Portalegre, Portugal; 8 Life Quality Research Center (LQRC-CIEQV), Complexo Andaluz, Apartado 279, 2001-904 Santarém, Portugal; 9 Department of Sports Sciences, University of Beira Interior, 6201-001 Covilhã, Portugal; 10 School of Sport and Health Sciences, Cardiff Metropolitan University, Cardiff CF5 2YB, UK; rmorgans@cardiffmet.ac.uk; 11 Department of Sports Sciences, Universidad Autónoma de Madrid (UAM), 28049 Madrid, Spain; 12 Department of Sports, Exercise and Health Sciences, University of Trás-os-Montes e Alto Douro, 5001-801 Vila Real, Portugal; 13 Department of Physical Culture and Sports, Urgench State University, Urgench 220100, Uzbekistan; nemat.r@urdu.uz

**Keywords:** wearable devices, tracking technologies, workload, monitoring, intensity, youth

## Abstract

Utilizing techniques for reducing multivariate data is essential for comprehensively understanding the variations and relationships within both biomechanical and physiological datasets in the context of youth football training. Therefore, the objective of this study was to identify the primary factors influencing training sessions within a standard microcycle among young sub-elite football players. A total of 60 male Portuguese youth sub-elite footballers (15.19 ± 1.75 years) were continuous monitored across six weeks during the 2019–2020 in-season, comprising the training days from match day minus (MD-) 3, MD-2, and MD-1. The weekly training load was collected by an 18 Hz global positioning system (GPS), 1 Hz heart rate (HR) monitors, the perceived exertion (RPE) and the total quality recovery (TQR). A principal component approach (PCA) coupled with a Monte Carlo parallel analysis was applied to the training datasets. The training datasets were condensed into three to five principal components, explaining between 37.0% and 83.5% of the explained variance (proportion and cumulative) according to the training day (*p* < 0.001). Notably, the eigenvalue for this study ranged from 1.20% to 5.21% within the overall training data. The PCA analysis of the standard microcycle in youth sub-elite football identified that, across MD-3, MD-2, and MD-1, the first was dominated by the covered distances and sprinting variables, while the second component focused on HR measures and training impulse (TRIMP). For the weekly microcycle, the first component continued to emphasize distance and intensity variables, with the ACC and DEC being particularly influential, whereas the second and subsequent components included HR measures and perceived exertion. On the three training days analyzed, the first component primarily consisted of variables related to the distance covered, running speed, high metabolic load, sprinting, dynamic stress load, accelerations, and decelerations. The high intensity demands have a high relative weight throughout the standard microcycle, which means that the training load needs to be carefully monitored and managed.

## 1. Introduction

Techniques for reducing multivariate data have recently been applied to analyze the training load variations and relationships in youth football. An understanding of the fluctuations in the training load throughout the weekly microcycle enables the more effective management of the training intensity in relation to technical–tactical objectives, player readiness and performance [1,2]. Nevertheless, the training planning, scheduling and periodization can be influenced by several key factors, including the expertise level, player status (such as starters versus non-starters), biological age, maturation status, relative age, playing position, session duration, and playing time [3,4,5]. Thus, understanding how these training loads vary throughout each training day is critical to fit a standard microcycle.

With training monitoring strategies, a large amount of information can be obtained quickly about training demands during the weekly microcycle. The most commonly used methods include wearable devices, tracking systems, perception of effort, heart rate (HR) telemetry, biomarker analysis, and performance data [6]. Tracking methods utilize devices such as global navigation satellite systems (GNSSs) and/or micro-electro-mechanical systems (MEMSs) to monitor the external training load during training sessions [7,8], providing high-intensity metrics such as the distance covered in different intensity zones, body impacts and accelerations [9]. Otherwise, the subjective perceived effort and heart rate (HR) monitors provide the internal training load [10,11]. Therefore, techniques for reducing multivariate data have been applied to extract the best information for the training and decision-making process. The principal component approach (PCA) has been described as an effective strategy for analyzing key indicators to simplify the complexity of multifactorial data [12,13]. Recently, PCA datasets were reduced by 75.07%, providing 3.9 ± 2.53 components that accounted for 80 ± 0.14% of the total explained variance in football insights [13].

Previously, the standard weekly training load has been scrutinized in elite and sub-elite youth football. Maughan et al. [14] identified correlations between internal and external training load measurements that showed within-individual relationships in 20 youth footballers. For training conducted three days before the match, three components (80.2% of variation) were kept, while two components were omitted (72.9–89.7% of variance). In another study, by Coppus et al. [15], the weekly analysis of the influencing factor of the training load identified the session rating of the perceived exertion as the variable that most correlated the training load with the external training load. Also, Scantlebury et al. [16] described that the external training variables were reduced to two PCs for each sport, cumulatively explaining 91% of the sRPE variance. The measures of volume (total distance, player load, and low-intensity running) were used to represent the first component, while the measures of intensity were used to characterize the second and third components [14]. Otherwise, Nosek et al. [17] described that two principal components were retained, explaining a total of 81% of the data variance. In their study, the first component comprised variables associated with distances in speed zones and the second component variables related to changes in direction.

Recently, the standard microcycle in young sub-elite footballers was analyzed by five principal components explaining 68.7% of the variance: (1) explosiveness, accelerations, and impacts (27.4%); (2) high-speed running (16.2%); (3) HR-based measures (10.0%); (4) baseline characteristics (8.3%); and (5) average running velocity (6.7%) [12]. The key factors for training load monitoring in sub-elite youth football were high-intensity variables with a strong relationship to maturational status [12,13]. However, the previous studies only considered the gross demands of the entire weekly training microcycle. From the point of view of planning and training, it is important to differentiate the preponderance of these physical demands according to the training day [18]. Despite this previous evidence, when reviewing the current literature, we identified that a PCA-based approach to differentiating the most preponderant physical subdominant was still not applied for each microcycle day (a shorter time line). Thus, this study aims to extract and reduce the biomechanical and physiological datasets from a standard weekly microcycle in young sub-elite football using a PCA approach.

## 2. Materials and Methods

### 2.1. Sample

This study involved sixty male sub-elite Portuguese soccer players, characterized by the following attributes: average height of 1.74 ± 0.08 m, weight of 62.48 ± 10.03 kg, body mass index (BMI) of 20.61 ± 2.14 kg/m^2^, sitting height of 88.36 ± 8.51 cm, predicted adult height of 14.20 ± 1.39 cm, average experience of 6.76 ± 1.42 years, and relative age of 0.25 ± 0.18 years. The young football players belonged to the U15 age group (*n* = 20), U17 (*n* = 20), and U19 (*n* = 20) of a Portuguese sub-elite football academy. These players were also grouped based on their stages of peak height velocity (PHV), which are Pre-PHV (*n* = 52 observations), Mid-PHV (*n* = 65 observations), and Post-PHV (*n* = 207 observations). Additionally, the players were classified according to their playing positions on the field. The number of observations for each position role included central defenders (CD, *n* = 79), fullbacks (FB, *n* = 65), central midfielders (CM, *n* = 70), wide midfielders (WM, *n* = 62), and forwards (FW, *n* = 48). The average experience for each group was 4.82 ± 0.90 years, 6.64 ± 1.65 years, 8.81 ± 1.70 years from the U15, U17 and U19 groups, respectively. All the participants received comprehensive information regarding the purpose and potential risks of the research according to ethical standards. Also, an informed consent form was signed by the parents and/or legal guardians of the young footballers. The study protocol was approved by the local ethics committee of the University of Trás-os-Montes e Alto Douro (3379-5002PA67807).

### 2.2. Study Design

The weekly training load was consistently evaluated, spanning a six-week duration comprising a total of 18 training sessions and 324 observation instances (2019–2020 competitive season). This period coincided with a schedule featuring one competitive game per week, with training sessions conducted three times weekly, each lasting approximately 90 min. These training sessions were integral to the dataset, with no inclusion of match data analysis. The training schedule adhered to a “match day minus format” (MD), with sessions scheduled on Tuesday, Wednesday, and Friday corresponding to MD-3, MD-2, and MD-1, respectively. On average, eighteen players attended each training session, which were conducted between 10:00 AM and 8:00 PM. These sessions took place on official-sized synthetic turf pitches (100 × 70 m), meeting FIFA standards, and occurred under similar ambient conditions, with temperatures ranging from 14 to 20 °C and relative humidity between 52% and 66% [3,4,5].

### 2.3. Selection Criteria

The eligibility of the training data was determined based on the following inclusion criteria: (a) participants had to be youth football players between the ages of 13 and 20, corresponding to the U15, U17, and U19 categories; (b) players were required to have at least 5 years of competitive experience in football; (c) the training sessions needed to contain at least 35 uninterrupted minutes of play, without any interruptions due to injury, withdrawal, or other non-regular reasons; and (d) the training data should correspond to a schedule of one competitive match per week with three training sessions. Data were excluded under the following conditions: (a) if players were absent, injured, ill, or had withdrawn during monitored sessions; (b) if players were engaged in separate activities due to rehabilitation, additional, or individual training sessions; and (c) if match data were not included in the analysis [12,14,19].

### 2.4. Standard Microcycle

**Figure 1:**
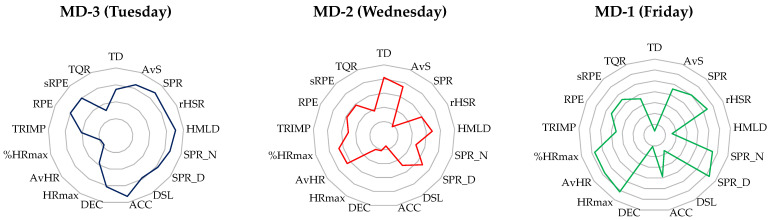
Weekly training overview of the sample sub-elite youth football academy. Path graphs from the principal component approach (PCA) on each training day (i.e., MD-3, MD-2 and MD-1) and weekly overview (i.e., Wk). Abbreviations: ACC—acceleration; AvHR—average heart rate. AvS—average speed; DEC—deceleration; HMLD—high metabolic load distance; HRmax—maximal heart rate; MD—match day; SPR_D—average sprint distance; SPR_N—number of sprints; sRPE—session ratings of perceived exertion; TD—total distance; TQR—total quality recovery; TRIMP—training impulse; %HRmax—percentage of maximal heart rate.

The collection of training data spanned a period of six weeks, encompassing a total of eighteen training sessions, equivalent to 324 observation instances, without including any analysis of match data. The criteria for dataset eligibility stipulated that participants were required to complete full training sessions lasting approximately 90 min, occurring three times per week, aligned with a competitive schedule of one game weekly. Training days were categorized using a “match day minus format” (MD), with Tuesday designated as MD-3, Wednesday as MD-2, and Friday as MD-1. On average, eighteen players participated in the training sessions held on an outdoor synthetic turf pitch measuring 100 by 70 m. A synthesis of the weekly training sessions at sub-elite youth football academies was compiled, considering various criteria, such as the training objectives, session duration, pitch dimensions, tactical objectives, numerical considerations (e.g., player per team), physiological goals emphasizing speed, agility, and quickness (SAQ), and specific training assignments and drills [12]. On Tuesday, the session is geared toward recovery and the refinement of technical skills. Players train on a half field (50 × 60 m) for 90 min at a moderate intensity, maintaining their exertion at 75–80% of their maximum heart rate (HRmax). Wednesday’s session shifts to acquisitive training, concentrating on game principles and collective behavior from a full field (100 × 60 m), pushing players to a higher intensity level of 90–95% of their HRmax. On Friday, the focus shifts to finishing and tactical execution in a half field (50 × 60 m), working at a high intensity, exceeding 85% of their maximum running speed [12,20]. This summary was formulated through the analysis of field notes and collaboration with the investigated sub-elite football academy. Additionally, the structure of the standard microcycle was constituted by small-sided games (SSG), medium-sided games (MSG), large-sided games (LSG) and SAQ training, including a range of scenarios from individual efforts (1 × 0 + Goalkeeper) to more complex team setups (11 × 0 + Goalkeeper), enhancing players’ ability to score from various positions and under different conditions [20,21]. Figure 1 illustrates the mean weekly training load in the sub-elite football academy.

### 2.5. Methods

#### 2.5.1. Individual Characteristics

Data on the players’ height (measured in meters), weight (measured in kilograms), chronological age (measured in years), sitting height (measured in centimeters), experience level (measured in years), and body mass index (BMI) were gathered. The calculation of relative age (a.u.) involved dividing the difference between the player’s birthdate and the deadline, which is 31 August, by the total number of days in a year, which is 365 days [22,23]. Maturity status was determined using the predicted adult height (PHV) and the predictive Mirwald’s equations [24]. Also, the detailed categorization was presented across different ages, maturational statuses, positional roles and experience levels.

#### 2.5.2. Wearable and Tracking Systems

The monitoring of sub-elite football players during training sessions involved the use of an 18 Hz global positioning system (GPS) in conjunction with an accelerometer (100 Hz), a magnetometer (10 Hz), and a gyroscope (100 Hz) (STATSports Apex^®^, Newry, Northern Ireland). A custom-made vest was worn by each player, positioned between their two scapulae to securely hold the GPS device and ensure optimal satellite signal reception for tracking purposes. All the devices were activated thirty minutes prior to the data collection [25,26]. The GNSSs are recognized as reliable tools for performance analysis in team sports, including football, despite some noted inaccuracies in terms of both linear and curved movements in GPS systems. The heart rate (HR) was monitored using a Garmin TM Heart Rate Band equipped with a 1 Hz short-range telemetry device (International Inc., Olathe, KS, USA) [27,28]. The perceived exertion levels were evaluated using the Portuguese 15-point Borg Rating of Perceived Exertion 6–20 Scale (Borg RPE 6–20) and the Total Quality Recovery (TQR) score, with individual data collected before and approximately 30 min after each training session [10]. The players were familiar with the protocols, and their perceived exertion levels were recorded using a Microsoft Excel^®^ spreadsheet (Microsoft Corporation, Albuquerque, NM, USA).

#### 2.5.3. External Training Load

The external training load was assessed through time–motion data, including metrics such as the total distance covered (TD) in meters, average speed (AvS), maximal running speed in meters per second (ms^−1^) (SPR), relative high-speed running (rHSR) distance in meters, high metabolic load distance (HMLD) in meters, sprinting (SPR_N) distance in meters, number of sprints (SPR_N), dynamic stress load (DSL) measured in arbitrary units (a.u.), number of accelerations (ACC), and number of decelerations (DEC). The GPS software provided information specifically on locomotor categories above 19.8 km/h: rHSR (19.8–25.1 km/h) and SPR (>25.1 km/h). The HMLD, a metabolic variable, was defined as the distance covered by a player when the metabolic power exceeds 25.5 W/kg, encompassing high-speed running and accelerations and decelerations above 3 ms^−2^ [29,30]. Both acceleration variables (ACC/DEC) accounted for movements in the maximum intensity zone (>3 ms^−2^ and <3 ms^−2^, respectively). The DSL was evaluated using a 100 Hz tri-axial accelerometer integrated into the GPS devices, measuring the sum of accelerations in the X, Y, and Z planes to calculate a composite magnitude vector expressed as the G force. The high-intensity activity thresholds were adapted from previous studies [7,8].

#### 2.5.4. Internal Training Load

HR-based measures including the maximum heart rate (HRmax), average heart rate (AvHR), and percentage of HRmax (%HRmax) were considered for analysis. The training impulse was calculated using the Akubat TRIMP, which reports a team TRIMP based on the individual training load from players’ iTRIMP. The Akubat TRIMP was calculated using the following equation: training duration × 0.2053e3.5179x, where e is the Napierian logarithm, 3.5179 is the exponent, and x is the HRratio [11]. The HRmax was obtained from the Yo-Yo intermittent recovery test level 1 (YYIR1) [9]. The perceived exertion was assessed using the 15-point Portuguese Borg Rating of Perceived Exertion 6–20 Scale (Borg RPE 6–20). The session rating of perceived exertion (sRPE) was obtained by multiplying the total duration of the training sessions by each individual’s RPE score. To monitor recovery, players reported their TQR score on a scale from 6 to 20, assessing the athletes’ recovery perceptions. The RPE and TQR scores were collected individually approximately 30 min after and before each training session, respectively, with data recorded using a Microsoft Excel^®^ spreadsheet [3,4,5].

### 2.6. Statistical Analysis

The training load variables influencing each training day of the typical weekly microcycle were identified through a data reduction technique. Initially, the presence of multicollinearity among the variables was assessed using Pearson’s parametric correlation (r). Subsequently, factor analysis was conducted utilizing the principal component approach (PCA), following the standard approach for monitoring data in team sports [31,32]. The determination of the number of extraction factors was based on an eigenvalue analysis, alongside a scree plot and Monte Carlo simulation. The Z scores were computed using Bartlett’s sphericity and the Kaiser–Meyer–Olkin (KMO) values to scale and center the final PCA selection variables. The decision on the number of PCA factors to retain was guided by the scree plot and chi-squared test, considering eigenvalues greater than one [32]. The scree plot of the eigenvalues analyzed the training data and simulated data from the parallel analysis [31,32].

The factor component loadings were determined using orthogonal rotation with a VariMax method to address perpendicularity in the correlation matrix of variables of interest. Variables with correlations below 0.4 (r < 0.4) were selected for extraction [31]. When the KMO values are more than 0.6 and Bartlett’s sphericity is less than 0.05, factor analysis is deemed appropriate [33]. Taking into account eigenvalues greater than 1, the number of PCs to be kept was ascertained using the scree plot of the obtained factor eigenvalues [31,34]. The sample size was calculated by G*Power, Version 3.1.5.1 (Institut für Experimentelle Psychologie, Düsseldorf, Germany), with an effect size ß of 0.4, an α of 0.05, and a power of 0.8 (1 − ß). For the principal component approach (PCA), a minimum of 150 cases, or 5 to 10 cases per variable, has been recommended as a minimum sample size. The weekly training load included a total of 18 training sessions, 324 observations and 19 variables [33].

The results of the PCA were presented through a path analysis, with the weightings (eigenvectors) depicted in a 2D graphic. Data were reported as the mean ± standard deviation with 95% confidence intervals (95% CI). Statistical significance was set at *p* < 0.05. IBM SPSS Statistics for Windows, Version 27.0 and JASP software were utilized for the analysis.

## 3. Results

### 3.1. Data-Reduction Procedure and Eigenvalues

**Figure 2:**
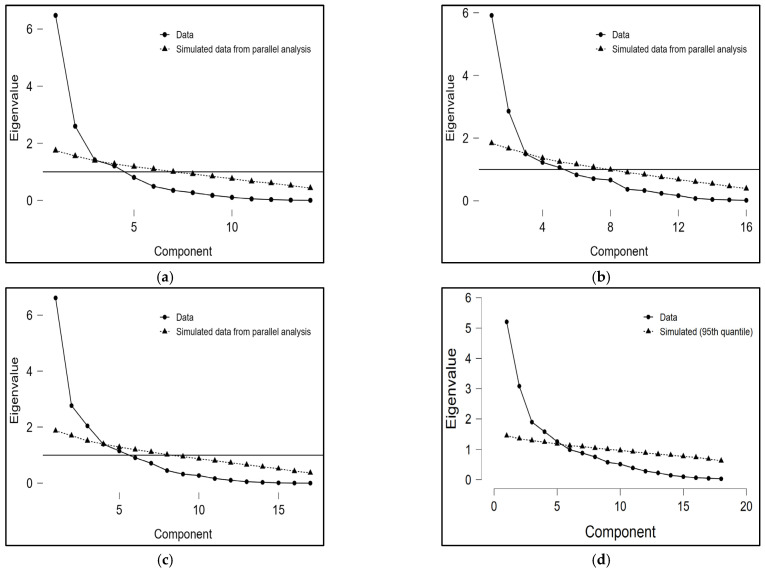
Scree plot of the eigenvalues between the data and the simulated data on MD-1 (**a**), MD-2 (**b**), MD-3 (**c**), and overall weekly microcycle (**d**).

The eigenvalues within the overall training data (i.e., weekly training load) ranged from 1.20% to 5.21%. Significant chi-squared values were observed for the PCA from MD-3 to MD-1 (*p* < 0.001). The biomechanical and physiological datasets were condensed into three to five principal components (PCs), which varied according to the training day. Analysis of the PCs based on the periodization structure revealed that the proportion and cumulative variance ranged from 12.0% to 38.9% (proportion) and 38.9% to 67.2% (cumulative) on MD-3. MD-2 exhibited a variance proportion and cumulative variance ranging between 17.0% and 37.0%, and 54.8%, respectively. On MD-1, the proportion and cumulative variance varied from 8.6% to 46.3% and 46.3% to 83.5%, respectively. Figure 2 shows the scree plot of the eigenvalues, analyzing the training data and simulated data from the parallel analysis on MD-1 (a), MD-2 (b), MD-3 (c), and overall week (d).

### 3.2. Principal Components

The PCA analysis identified distinct key components for each training day of the standard microcycle. In the MD-3, MD-2, and MD-1 phases, the first component primarily included the TD, AvS, HMLD, SPR, DSL, ACC, and DEC. The second component in these phases comprised HR measures and the TRIMP. Additionally, the MD-1 phase included the third to fifth components involving the AvS, sRPE, and TQR scores. In the Wk phase, the first component again incorporated variables related to the distance covered, HMLD, DSL, ACC, and DEC, while the second component included HR measures and the TRIMP. The third to fifth components in the week phase encompassed the AvS, SPR, sRPE, and TQR scores.

**Figure 3:**
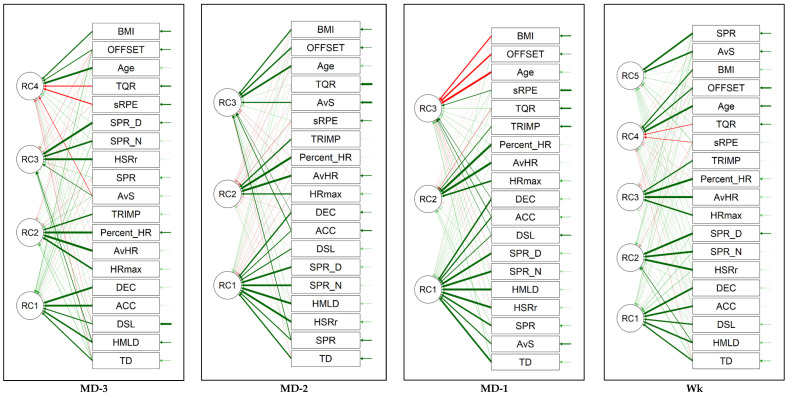
Path graphs from the principal component approach (PCA) on each training day (i.e., MD-3, MD-2 and MD-1) and weekly overview (i.e., Wk). Abbreviations: ACC—acceleration; AvHR—average heart rate; AvS—average speed; BMI—body mass index; DEC—deceleration; DSL—dynamic stress load; HMLD—high metabolic load distance; HRmax—maximal heart rate; MD—“match day minus format” (MD); HSRr—relative high speed running; Percent_HR—percentage of maximal heart rate; RC—reduced component; SPR—average sprint distance; SPR_N—number of sprints; sRPE—session ratings of perceived exertion; TD—total distance; TQR—total quality recovery; TRIMP—training impulse; Wk—week.

Analyzing each training day, the MD-1, rHSR (m), ACC (m·s^−2^), and DEC (m·s^−2^) have the highest influence. The SPR (*n*) and DSL (au) are less influential compared to the other training days. On MD-2, the SPR (*n*), rHSR (m), HMLD (m), and SPR (m) stand out as highly influential variables, with coefficients around 0.90 or higher. The ACC (m·s^−2^) and Vmax (m·s^−1^) are less influential in this component. For MD-3, variables like the rHSR (m), HMLD (m), SPR (*n*), and SPR (m) have high coefficients close to or above 0.86, indicating they are the most influential in the first principal component. The variables like the DSL (au) and ACC (m·s^−2^) have lower coefficients, suggesting they are less influential. Across the week, the ACC (m·s^−2^) and DEC (m·s^−2^) are the most influential, with coefficients of 0.84 and 0.88, respectively. The TD (m) and HMLD (m) also contribute significantly. Figure 3 illustrates the outcomes of the PCA and the weightings (eigenvectors) through a path graph and a 3D plot. The equations derived from the extracted principal components are detailed in Table 1. The table lists the principal components and their associated variables, along with their coefficients for the different measurement days (MD-3, MD-2, and MD-1) and the week (Wk). The coefficients represent the weight of each variable in the principal component. The magnitude of these coefficients indicates the contribution of each variable to the principal component.

## 4. Discussion

This study aimed to identify the primary influencing factors on training days within a standard microcycle for young sub-elite football players by employing the PCA. The training datasets were condensed into three to five principal components, explaining between 37.0% and 83.5% of the variance (proportion and cumulative) according to the training day. The findings revealed variations in the most common biomechanical and physiological variables across the microcycle’s training days, emphasizing the importance of considering the partial weighting of each variable in the weekly periodization. Additionally, the high-intensity demands have a significant relative weighting throughout the standard microcycle, necessitating the careful monitoring and management of the training load.

### 4.1. Training Day Analysis

The PCA revealed distinct components influencing the training load variables on MD-3, MD-2, and MD-1. The first component consistently encompassed variables related to the distance covered, running speed, high metabolic load, sprinting, dynamic stress load, accelerations, and decelerations. This suggests that these variables collectively contribute to the overall training stimulus experienced by players during these phases. The second component predominantly included HR measures and the TRIMP, reflecting the physiological strain imposed on players during training sessions [35,36]. Interestingly, in the MD-1 phase, additional components (third to fifth) emerged, incorporating the average speed, sRPE, and TQR scores [3]. This highlights the importance of subjective measures and recovery perceptions in assessing the training load toward the end of the microcycle [4,12].

The resultant equations were derived from the PCA conducted for the different training days (MD-3, MD-2, MD-1) and the weekly microcycle (Wk). Each PCA analysis reveals the variables involved and the corresponding calculation for each principal component. For instance, on MD-3, the first component includes variables such as the TD, AvS, Vmax, and others, with specific weightings assigned to each variable in the calculation. Similar equations are provided for subsequent principal components and other training days. The PCA was applied to the external training intensity variables across different phases of the microcycle [4,12]. Each row represents a specific variable, while the columns correspond to different principal components (PCA 1 to PCA 5). The values within the table indicate the factor component loadings (eigenvectors) for each variable in each principal component. For example, in the MD-1 phase, the TD has the highest weighting load for PCA 5, suggesting its varying contribution to different components across training days. When comparing these findings with the existing literature, several parallels and distinctions emerge, offering deeper insights into the management of training loads in youth football. This study identified correlations between internal and external training load measurements in youth footballers. For training conducted three days before the match, three components explained 80.2% of the variance. For the other days, two components captured 72.9% to 89.7% of the variance [37]. The first component included measures of volume (total distance, player load), while the second and third captured intensity metrics, similar to findings where distance and speed metrics were predominant in the first components. The session rating of perceived exertion (sRPE) was found to be highly correlated with the training load, highlighting the significance of perceived effort in assessing the external training load. This is consistent with the inclusion of the sRPE in this study, particularly in the later components for MD-1 and the Wk phase [15]. In Scantlebury et al.’s study, the external training load variables were reduced to two principal components, explaining 91% of the variance in the sRPE. The first component was dominated by volume measures, while the second focused on intensity. This study’s emphasis on the TD, HMLD, and ACC in the primary components aligns with this focus on volume and intensity measures [16]. In the study by Nosek et al. [17], two principal components were retained, explaining 81% of the data variance. The first component was associated with distance metrics in the speed zones and the second with changes in direction, which resonates with the present findings, where the ACC and DEC were key variables, particularly on MD-1 and the weekly overview [17].

### 4.2. Weekly Analysis

The extracted principal components elucidated the relationships among the biomechanical and physiological variables across different training days. The first PC highlighted velocity, acceleration, and impact measures, while the second PC encapsulated all HR-based measures, underlining the physiological demands of the weekly training load [38]. Additionally, the perceived recovery, exertion, mean speed, and top speed formed a relationship across the extracted principal components, suggesting that the perceived exertion may be influenced by variables associated with the trainability, maturation, and stage of development [5,12]. The composite equation derived from this study can be utilized to measure biomechanical and physiological datasets from a standard weekly microcycle at a sub-elite youth football academy [31,32].

The current weekly analysis describes a comprehensive understanding of the underlying structure of the training load variables and their relationships across different phases of the microcycle. The resultant equations offer specific insights into the composition of the principal components and the weighting of the variables within each component [39,40]. A previous PCA analysis in sub-elite football training analyzed only the gross demands of the weekly training load, having identified 68.7% of the variance, covering aspects like explosiveness, accelerations, impacts (27.4%), high-speed running (16.2%), HR-based measures (10.0%), baseline characteristics (8.3%), and average running velocity (6.7%) [12]. The principal component approach (PCA) identified the key influencing variables for training sessions. The first component, comprising the total distance (TD), high metabolic load distance (HMLD), dynamic stress load (DSL), accelerations (ACC), and decelerations (DEC), weighted heavily on the DEC (0.88), ACC (0.84), and HMLD (0.79). The second component focused on heart rate measures, with the average heart rate (AvHR) having the highest weight (0.96), followed by the percentage of maximum heart rate (%HRmax) (0.95). The third component included the average speed (AvS), maximum velocity (Vmax), session rating of perceived exertion (sRPE), and total quality recovery (TQR), with these variables listed without specific weights.

By synthesizing the information from both tables, practitioners can gain valuable insights into the key determinants of the training load and its variability across different training days and the weekly microcycle [41]. This understanding can inform designs concerning more targeted and effective training programs tailored to the specific needs and goals of athletes, ultimately enhancing performance and reducing the risk of injury [42,43]. Additionally, it facilitates the monitoring and adjustment of training loads to optimize athletes’ readiness and recovery throughout the microcycle [44]. This discrepancy may be attributed to the sub-elite youth football context of this study compared to previous studies focusing on elite contexts. Previous research primarily utilized the PCA as a data reduction technique for match data analysis, with some attempts made to establish links between internal and external variables in professional adult and youth football players [12].

In sub-elite youth football, the age-related influences on the external and internal training load, as well as the recovery status, emphasize the importance of considering the developmental stage when designing training regimens [3]. While the playing position minimally impacts the weekly load, the inter-day and inter-week variations underscore the need for adaptable programming. Furthermore, the interaction effects observed between age, maturity status, and relative age necessitate a nuanced understanding of individual player characteristics to optimize training outcomes [45]. Consequently, an ideal training week should flexibly integrate these factors to ensure personalized and effective development strategies for sub-elite youth footballers [46]. Thus, the first component seems to be more related to external load indicators, the second component to external load indicators and the third and fourth components to individual and perceptual characteristics. These results are in line with previous composite equations published for young sub-elite footballers [17].

### 4.3. Practical Applications, Limitations and Future Directions

These findings have several practical implications for coaches and practitioners involved in training load monitoring in sub-elite youth football. Firstly, understanding the principal components across the weekly microcycle can help tailor training programs to optimize players’ readiness, performance and recovery. Coaches can adjust the training load based on the identified components to ensure a balanced and progressive training stimulus throughout the exercises, sessions, and weeks. Secondly, valuable insights into players’ readiness and recovery status can be gained by incorporating subjective measures such as the sRPE and TQR scores toward the microcycle. Understanding the dominant variables helps in designing training programs that focus on the most critical aspects of performance. This helps in making informed decisions about training, monitoring, and managing athletes, guiding decisions regarding the training intensity and volume in the next macrocycle, and thus, acquiring the property of changing the nature of the next weekly phase based on more recent biological changes in players’ physiology [47]. More importantly, defining the main components is a way of standardizing the microcycle for a particular team, training process and, above all, the intra- and inter-individual training load variations throughout the season phases (i.e., pre-season, competition, taper) [48,49].

Also, it is important to acknowledge some research limitations. The sample size and duration of the data collection may limit the generalizability of the findings to the whole population. Additionally, while the PCA provides a comprehensive approach to analyzing training load data, other statistical techniques such as cluster analysis or PCA-based machine learning (ML) algorithms over classical statistical approaches [48,49,50] could offer complementary insights. Specifically, it would be interesting to apply supervised and unsupervised machine-learning models in order to expand the current evidence to automated forms of analysis and information extraction in training load monitoring [50]. Also, future research could explore the longitudinal effects of the training load components on performance and injury risk in sub-elite youth football players, considering individual differences in maturation status and playing position. Also, future studies should consider the effects of the SAQ, shortening–stretching cycle (SSC), and plyometric training on mental and physical fatigue, as well as the influence of contextual variables and other performance dimensions such as technical and tactical factors [51,52]. Furthermore, future research should examine the combined covariate effect of the independent variables, considering factors such as the starting status, maturational outsets, positional roles, task design, and contextual factors [8]. Rather than the physiological set definition, this can have more to do with the training day’s tactical goal [41]. Thus, when planning and periodizing the weekly training, coaches, sports scientists, and athletes should take into account the weighting of each biomechanical and physiological aspect. Additionally, it is important to consider how the main ones vary according to the task design, considering the scales and tactical dimensions [53,54].

## 5. Conclusions

In conclusion, this study utilized the PCA to identify key factors influencing training sessions within a standard microcycle for young sub-elite football players, revealing that internal and external training vary significantly across different training days. The principal components explained between 37.0% and 83.5% of the variance, highlighting the importance of considering each variable’s weighting in the weekly periodization. These findings emphasize the need for careful monitoring and management of high-intensity demands throughout the standard microcycle to optimize players’ performance and recovery.

## Figures and Tables

**Table 1:** Resultant equations from analysis of the extracted principal components for the training days and weekly microcycle.

	PCA	Variables	Calculation
MD-3	1	TD (m), AvS (m·min^−1^), SPR (m·s^−1^), rHSR (m), HMLD (m), SPR_N (*n*), SPR_D (m), DSL (au), ACC (m·s^−2^), DEC (m·s^−2^)	0.81 × TD (m) + 0.62 × AvS (m·min^−1^) + 0.76 × SPR (m·s^−1^) + 0.90 × rHSR (m) + 0.90 × HMLD (m) + 0.89 × SPR_N (*n*) + 0.86 × SPR_D (m) + 0.53 × DSL (au) + 0.67 + ACC (m·s^−2^) + 0.75 × DEC (m·s^−2^)
	2	HR_max_ (bpm), AvHR (bpm), %HR_max_, TRIMP (au)	0.78 × HR_max_ (bpm) + 0.94 × AvHR (bpm) + 0.94 × HR_max_ (%) + 0.52 × TRIMP (au)
	3–5	TQR (au)	0.51 × TQR (au)
MD-2	1	TD (m), V_max_ (m·s^−1^), rHSR (m), HMLD (m), SPR_N (*n*), SPR_D (m), DSL (au), ACC (m·s^−2^), DEC (m·s^−2^)	0.76 × TD (m) + 0.55 × SPR (m·s^−1^) + 0.92 × rHSR (m) + 0.93 × HMLD (m) + 0.94 × SPR_N (*n*) + 0.90 × SPR_D (m) + 0.63 × DSL (au) + 0.46 + ACC (m·s^−2^) + 0.75 × DEC (m·s^−2^)
	2	HR_max_ (bpm), AvHR (bpm), %HR_max_, TRIMP (au)	0.72 × HR_max_ (bpm) + 0.95 × AvHR (bpm) + 0.95 × HR_max_ (%) + 0.71 × TRIMP (au)
	3–5	AvS (m·min^−1^)	0.67 × AvS (m·min^−1^)
MD-1	1	TD (m), rHSR (m), HMLD (m), SPR_N (*n*), DSL (au), ACC (m·s^−2^), DEC (m·s^−2^)	0.64 × TD (m) + 0.92 × rHSR (m) + 0.76 × HMLD (m) + 0.55 × SPR_N (*n*) + 0.61 × DSL (au) + 0.88 + ACC (m·s^−2^) + 0.90 × DEC (m·s^−2^)
	2	HR_max_ (bpm), AvHR (bpm), %HR_max_, TRIMP (au)	0.79 × HR_max_ (bpm) + 0.96 × AvHR (bpm) + 0.93 × HR_max_ (%) + 0.72 × TRIMP (au)
	3–5	AvS (m·min^−1^), sRPE (au), TQR (au)	–0.44 × AvS (m·min^−1^) − 0.60 sRPE (au) − 0.59 TQR (au)
Wk	1	TD (m), HMLD (m), DSL (au), ACC (m·s^−2^), DEC (m·s^−2^)	0.70 × TD (m) + 0.79 × HMLD (m) + 0.71 × DSL (au) + 0.84 + ACC (m·s^−2^) + 0.88 × DEC (m·s^−2^)
	2	HR_max_ (bpm), AvHR (bpm), %HR_max_, TRIMP (au)	0.77 × HR_max_ (bpm) + 0.96 × AvHR (bpm) + 0.95 × HR_max_ (%) + 0.69 × TRIMP (au)
	3–5	AvS (m·min^−1^), SPR (m·s^−1^), sRPE (au), TQR (au)	0.51 × AvS (m·min^−1^), SPR (m·s^−1^) − 0.46 × sRPE (au), − 0.42 × TQR (au)

Abbreviations: ACC—acceleration; AvHR—average heart rate; AvS—average speed; DEC—deceleration; DSL—dynamic stress load; HMLD—high metabolic load distance; HRmax—maximal heart rate; MD—“match day minus format”; rHSR—relative high speed running; SPR_D—average sprint distance; SPR_N—number of sprints; sRPE—session ratings of perceived exertion; TD—total distance; TQR—total quality recovery; TRIMP—training impulse; %HR—percentage of maximal heart rate.

## Data Availability

Data are available upon request to the contact author.
